# The WSB1 Gene Is Involved in Pancreatic Cancer Progression

**DOI:** 10.1371/journal.pone.0002475

**Published:** 2008-06-25

**Authors:** Cendrine Archange, Jonathan Nowak, Stéphane Garcia, Vincent Moutardier, Ezequiel Luis Calvo, Jean-Charles Dagorn, Juan Lucio Iovanna

**Affiliations:** 1 INSERM U.624, Stress Cellulaire, Parc Scientifique et Technologique de Luminy, Marseille, France; 2 Molecular Endocrinology and Oncology Research Center, CHUL Research Center, Sainte-Foy, Canada; Centre de Regulació Genòmica, Spain

## Abstract

**Background:**

Pancreatic cancer cells generate metastases because they can survive the stress imposed by the new environment of the host tissue. To mimic this process, pancreatic cancer cells which are not stressed in standard culture conditions are injected into nude mice. Because they develop xenografts, they should have developed adequate stress response. Characterizing that response might provide new strategies to interfere with pancreatic cancer metastasis.

**Methodology/Principal Findings:**

In the human pancreatic cancer cell lines Panc-1, Mia-PaCa2, Capan-1, Capan-2 and BxPC3, we used Affymetrix DNA microarrays to compare the expressions of 22.000 genes in vitro and in the corresponding xenografts. We identified 228 genes overexpressed in xenografts and characterized the implication of one of them, WSB1, in the control of apoptosis and cell proliferation. WSB1 generates 3 alternatively spliced transcripts encoding distinct protein isoforms. In xenografts and in human pancreatic tumors, global expression of WSB1 mRNA is modestly increased whereas isoform 3 is strongly overexpressed and isoforms 1 and 2 are down-regulated. Treating Mia-PaCa2 cells with stress-inducing agents induced similar changes. Whereas retrovirus-forced expression of WSB1 isoforms 1 and 2 promoted cell growth and sensitized the cells to gemcitabine- and doxorubicin-induced apoptosis, WSB1 isoform 3 expression reduced cell proliferation and enhanced resistance to apoptosis, showing that stress-induced modulation of WSB1 alternative splicing increases resistance to apoptosis of pancreatic cancer cells.

**Conclusions/Significance:**

Data on WSB1 regulation support the hypothesis that activation of stress-response mechanisms helps cancer cells establishing metastases and suggest relevance to cancer development of other genes overexpressed in xenografts.

## Introduction

Pancreatic cancer is a highly lethal disease with a poor long-term survival rate for patients with locally advanced and metastatic stages[1]–[3]. Major reasons for this poor outcome are our inability to diagnose pancreatic cancer at an early stage because of the lack of specific symptoms, location of the pancreas deep into the peritoneal cavity and early occurrence of metastases. Therefore, in the majority of patients, by the time of diagnosis, regional lymph node and liver metastasis of pancreatic cancer has occurred, limiting the effectiveness of the only known curative options which must include surgical resection[4]–[6]. To date, radiotherapy or chemotherapy have not resulted in significant improvement in long-term survival[7]. The causes of pancreatic cancer are not yet precisely understood. Many gene products show deregulated functions. Numerous growth factors and their receptors are overexpressed during the progression of pancreatic cancer[7]–[9] but these findings have hardly led to any improvement in pancreatic cancer therapy.

Our strategy to identify new potential targets for pancreatic cancer therapy is based on the idea that tumoral progression is always accompanied by changes in the microenvironment and that its success depends on the ability of cancer cells to adapt to this new microenvironment. In fact, it is known that cancer progression involves several successive steps. Within the primary tumor, cells are first selected on the basis of rapid growth, low response to apoptotic signals and a capacity to escape the host immune system. These cells leave the primary tumor and migrate through the organism. However, not all of them will generate metastases in all tissues. That process is selective and depends both on the capacity of tumor cells at establishing interactions with the host tissue and on their capacity at managing the environmental factors to which they are exposed in their new location. The phenotype of the tumor cell is adapted to its original environment, the environment of the organ in which the primary tumor developed. Since the environment provided by the target organ is different from the original one, it will put the invading tumor cell under microenvironmental stress pressure. As a consequence, the ability of the tumor cell to resist that stress could be determinant in the success of metastasis development. The cell will activate its defence program in order to survive and, if successful, proliferate. Our goal was to identify the genes involved in this program because inhibition of their expression might be a new way of targeting pancreatic cancer. In this paper we describe a panel of potential pancreatic cancer genes selected using a xenograft strategy and analyze the structure and function of one of them, the WSB1 gene, in relation to pancreatic tumor progression.

## Results

### The panel of pancreatic cancer stress genes

The model we chose to establish the panel of potential pancreatic progression-related genes is the following: We used 5 human pancreatic cancer cell lines with different genetic backgrounds, Panc-1, Mia-PaCa2, Capan-1, Capan-2 and BxPC3. We made the hypothesis that, in standard culture conditions, these cells are not stressed by their microenvironment. Upon injection into the nude mouse, they will be exposed to the stress of a different environment the xenograft mimicking metastasis. Although the xenograft model reflects only partly what really happens with metastasis, it is a good means to put cells in an environment quite different from their usual environment. As a consequence an important stress will be generated, to which the cells will have to resist. In each cell line, we compared the expressions of 22.000 genes in vitro and in the xenografts, using the Affymetrix microarray approach. To ensure that selected genes are representative of a large proportion of human pancreatic tumors, only genes whose expression was modified at least twofold in all 5 tumors were considered. Under these conditions, we could select 228 genes overexpressed in tumors and 13 which were down-regulated. The full data are accessible online at http://www.inserm-u624.univ-mrs.fr/affymetrix/. Several among the most overexpressed genes are poorly characterized if not totally unexplored. As a first step, we chose to focus on WSB1 (WD-repeat and SOCS Box containing-1) because, as described hereunder, it showed the original feature of generating, by alternative splicing, 3 variant transcripts encoding distinct protein isoforms, whose regulation in xenografts and upon exposure to several stresses is not coordinated.

### WSB1 expression in pancreatic tumor xenografts

**Figure 1:**
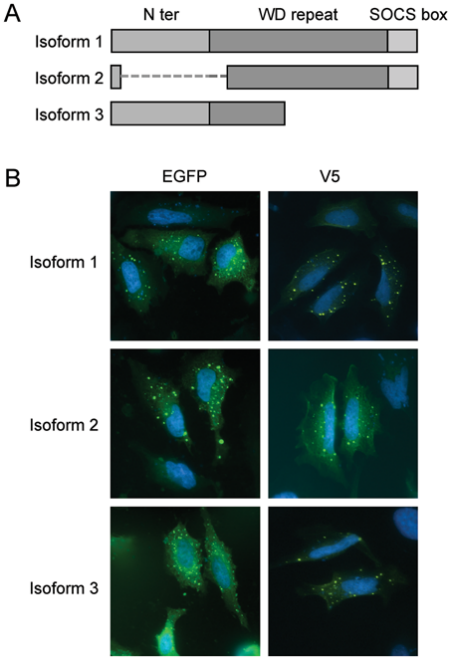
The three proteic isoforms of wsb1 and their subcellular localization. A. Schematic representation of WSB1 isoforms 1, 2 and 3. B. WSB1 isoforms were expressed as EGFP or V5 tag fusion proteins and subcellular localization was analyzed by direct fluorescence of after immunocytochemistry using EGFP or V5 antibodies respectively.

The complete protein generated by transcript variant 1, isoform 1, is composed of 3 domains (an N-terminal domain, a protein-protein interaction domain comprising 8 WD-repeats, and a SOCS box) (Figure 1). Isoform 2 lacks almost the entire N-terminal domain and the first WD-repeat. Finally, a premature stop codon in isoform 3 generates a truncated protein lacking the C-terminal part of the protein, corresponding to 6 WD-repeats and the SOCS box.

**Figure 2:**
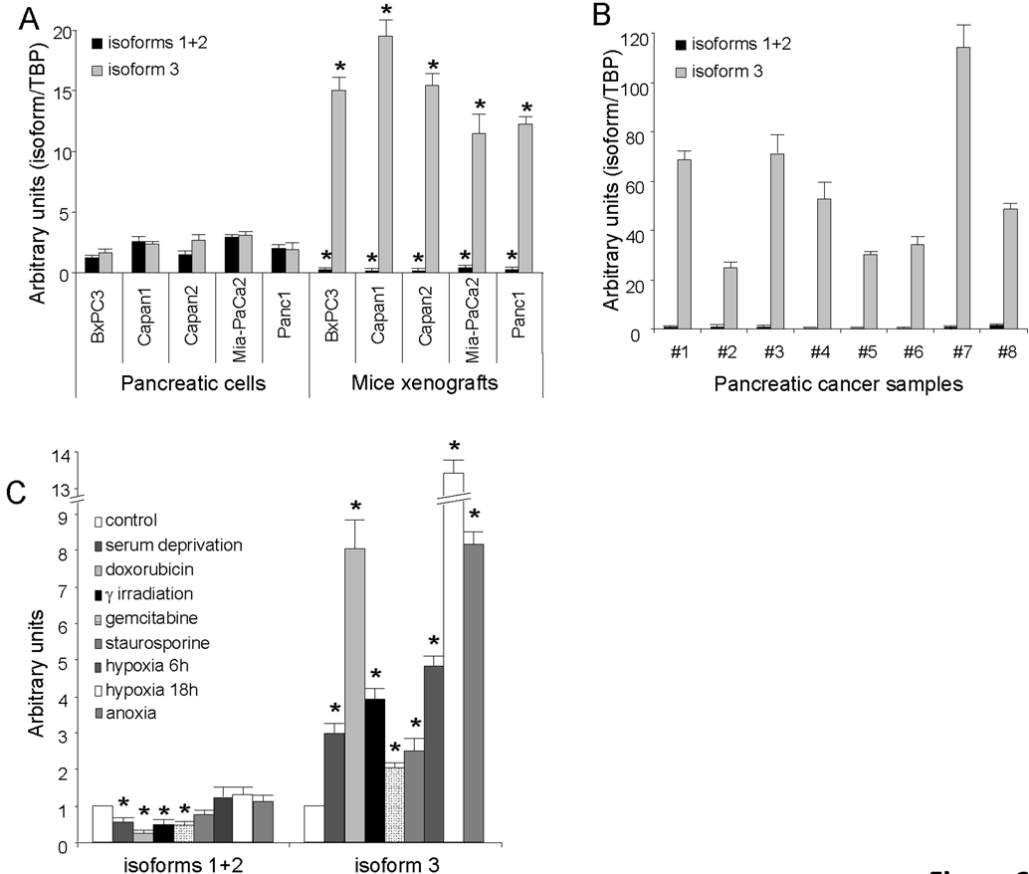
Splicing is in favor of isoform 3 in mice xenografts, human pancreatic tumors and after stress. Expression of WSB1 isoforms 1+2 and 3 mRNAs was assessed by qRT-PCR in pancreatic cancer cell lines and in mice xenografted tumors (A), in human pancreatic cancer samples (B), and in Mia-PaCa2 cells after treatment with stress-inducing agents (C). Values are expressed as the mean±S.D. of combined results from two independent experiments performed in triplicate. (*, p<0.05).

If global expression of the gene, detected by the 213406_at Affymetrix probe corresponding to the transcription of all three isoforms, is only increased 1.17 to 2.38 fold in mouse xenografts depending of the cell line, the expression of isoform 3, detected by the 201295_s_at Affymetrix probe, is increased 4.63 to 20.32 fold (Table 1). These results were controlled by quantitative RT-PCR analysis. We developed *Homo sapiens*-specific primers to analyze independently the expressions of WSB1 isoform 3, isoforms 1+2 and isoforms 1+2+3 (global expression) (Figure S1). We found that global expression of WSB1 was increased by 1.75 to 3.93 fold in tumors (data not shown). Interestingly, expression of isoform 3 was increased by 3.7 to 9 fold whereas that of isoforms 2+3 was decreased by 5 to 20% (Figure 2), in agreement with DNA chip data. Therefore, rather than an increase in WSB1 global expression in xenografts tumors, compared to cells maintained in vitro, we observed a significant shift in alternative splicing in favor of isoform 3 which becomes largely predominant in tumors.

**Table 1:** Microarray signal intensities recorded for WSB1 (arbitrary units)

		In vitro	Xenografts	ratio
201295_s_at Isoform 3	BxPC3	77.8	790.9	10.2
	Panc-1	114.9	532.0	4.6
	Capan-1	66.8	529.9	7.9
	Capan-2	33.9	688.9	20.3
	Mia-Paca2	75.0	407.6	5.4
213406_at Isoforms 1+2+3	BxPC3	97.7	158.6	1.6
	Panc-1	117.8	230.1	1.9
	Capan-1	109.2	144.2	1.3
	Capan-2	55.2	131.6	2.4
	Mia-Paca2	124.2	145.4	1.2

To check the clinical relevance of these findings, we measured the expression of the WSB1 isoforms by quantitative RT-PCR in 8 human pancreatic adenocarcinoma samples. We observed in these tumors a very strong predominance of isoform 3 expression with a low level of isoforms 1+2 (Figure 2). This suggests that the expression profile of WSB1 observed in mice xenografts is representative of what happens in human pancreatic cancer.

### Cellular stress modulates WSB1 alternative splicing in favor of isoform 3

Our hypothesis is that some of the genes over-expressed in tumors are activated in response to the stress caused by the new tissue microenvironment. In our experimental model, the environment of pancreatic cancer cells is indeed quite different when they are cultivated *in vitro* or growing in the *nude* mouse, in terms of nutrients, growth matrix, hormones, etc. Yet, some of these changes might be important enough to influence gene expression but not to the point of inducing stress. To check whether the observed changes in WSB1 gene expression are indeed a consequence of stress, we monitored the expressoin of WSB1 mRNA isoforms in Mia-Paca2 cells, in response to the stress generated by various treatments. These treatments affecting different pathways should also provide information on the function of WSB1. They included the induction of apoptosis with staurosporine, or adriamycine and gemcitabine which block DNA synthesis, DNA damage by γ-irradiation, metabolic stress by serum starvation and hypoxia or anoxia. As shown in Figure 2, all treatments resulted in a very modest change in the expression of WSB1 isoforms 1+2 and in a strong increase in isoform 3 expression. Therefore, WSB1 isoform 3 expression seems to be enhanced in response to stress, whatever the stress pathway involved. Although we cannot rule out that this is in part influenced by the presence of stroma in tumors, these results suggest that the changes in alternative splicing observed for WSB1 in our xenograft model are indeed modulated by cell stress. Similar results were obtained with Panc1 cells (data not shown).

### Subcellular localization of WSB1 isoforms 1, 2 and 3

To analyze the subcellular localization of WSB1 isoforms 1, 2 and 3 we subcloned the 3 cDNAs into the pEGFP-N1 vector to obtain WSB1 isoforms as EGFP-WSB1 fusion proteins. These plasmids were transfected into HeLa cells and after 24 h, isoforms 1, 2 and 3 appeared as cytoplasmic dots. No differences in dot size or number were found between the three isoforms. EGFP-WSB1 fusion proteins may acquire abnormal spatial conformations preventing them to reach the normal location of the protein and possibly inducing aggregate formation which could account for our observations. As a control, we subcloned the WSB1 isoforms into pcDNA4 to produce three WSB1-V5 tagged proteins. Plasmids were transfected into HeLa cells and anti-V5 antibodies used to detect 24 h later the isoforms by immunocytochemistry. Interestingly, the subcellular distribution of the V5-tagged isoforms was very similar to that observed for EGFP-WSB1 isoforms (Figure 1).

### WSB1 enhances cell proliferation

**Figure 3:**
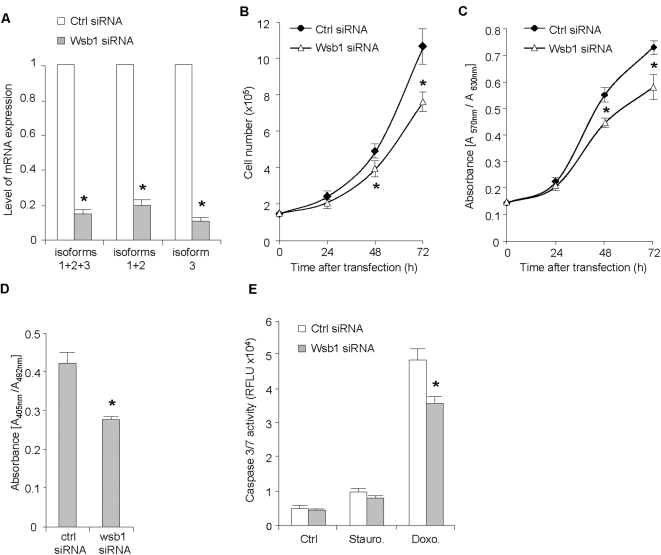
WSB1 enhances cell proliferation. A. Mia-PaCa2 cells were transfected with a siRNA directed against WSB1 mRNA and expression of WSB1 isoforms 1+2+3, 1+2 and 3 mRNAs was analyzed by qRT-PCR. B. Mia-PaCa2 cells were transfected with the WSB1 siRNA and growth was analyzed by direct cell counting (B), MTT analysis (C) or by BrdU incorporation (D) as described in Material and Methods section. E. Mia-PaCa2 cells were transfected with the WSB1 siRNA and caspase 3 activity was measured in untreated and after treatment with staurosporine or doxorubicin. Values are expressed as the mean±S.D. of combined results from two independent experiments performed in triplicate. (*, p<0.05).

Little is known about WSB1 function. WSB1, also known as SWIP1, was first identified by Vasiliauskas et al. [10] by searching human sequences similar to that of the chicken gene swip1. WSB1 shares 88% amino acid identity with the chicken swip1. The authors showed that swip1 was regulated by sonic hedgehog in somites and limb buds in the developing chicken. More recently, Dentice et al. [11] presented evidence that WSB1 is part of an E3 ubiquitin ligase for thyroid hormone-activating iodothyronine deiodinase-2. It is important to note that all previous experiments have been carried out in chicken where only two isoforms, corresponding to human isoforms 1 and 2 have been identified. Therefore, the function of this gene in human cancer, particularly of its isoform 3, is completely unknown. We first investigated the role of WSB1 on cell proliferation. To this end, we inhibited WSB1 expression by transfecting Mia-Paca2 with a siRNA that targets the 3 isoforms. As shown in Figure 3, 48 h after transfection, this siRNA efficiently reduced WSB1 global expression by 85% (80% for isoforms 1+2 and 89% for isoform 3). Cell growth was measured every 24 h, starting 48 h after siRNA transfection, by direct cell counting and by MTT assay. In both cases, results indicate that cells transfected with WSB1 siRNA grew more slowly than cells transfected with the control siRNA (Figure 3). Similar results were obtained with Panc1 cells (data not shown). BrdU incorporation experiments confirmed these results. However, that difference could be due to an increase in cell death rather than to a decreased proliferation of WSB1 siRNA-transfected cells. To test this hypothesis, we assessed apoptosis by measuring caspase-3 activity in cells transfected with these siRNA. Caspase-3 activity was the same in cells transfected with WSB1 siRNA or with control siRNA. Moreover, when apoptosis was induced by treatments with staurosporine or doxorubicin, cells transfected with WSB1 siRNA showed a lower caspase-3 activity than control cells (Figure 3). Taken together, these results strongly suggest that WSB1 expression enhances cell growth by increasing cell proliferation rather than by decreasing cell death.

### A specific role for isoform 3

**Figure 4:**
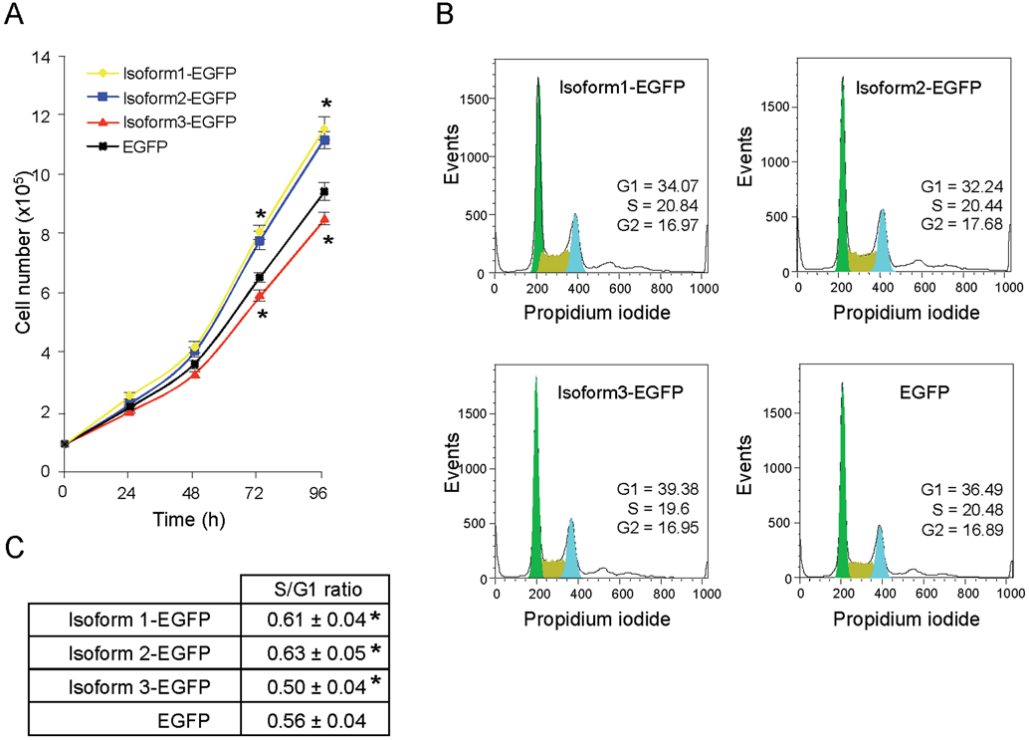
A specific role for isoform 3 which reduces pancreatic cancer cell growth. A. Mia-PaCa2 cells were transduced with retrovirus expressing WSB1 isoforms 1, 2 or 3 as EGFP fusion protein or EGFP alone as a control and selected with puromycin. Cell growth was measured by direct counting after plating 10^5^ cells in 48-well dishes every day during 4 days. B. After synchronization with 24 h serum-starvation, cells were incubated in serum containing medium for 24 h, stained with propidium iodide and cell cycle was analysed with a flow cytometer. C. S/G1 ratio. Values are expressed as the mean±S.D. of combined results from two independent experiments performed in triplicate (A) or trice in triplicate (B and C) (*, p<0.05).

Expression of WSB1 isoform 3 is induced in human pancreatic cancer cells both in vitro after exposure to a stress and in vivo when these cells grow as xenografts in mice. It is also largely predominant in human pancreatic tumors, suggesting that the 3 isoforms have different functions. To characterize these functions we generated stable cell lines expressing each isoform of WSB1 separately as WSB1-EGFP fusion proteins, by transducing Mia-PaCa2 cells with retrovirus encoding WSB1 isoforms 1, 2 or 3. Expression of fusion proteins was evidenced by western blot (Figure S2). We measured cell growth by direct counting each 24 h during 4 days and found that cells expressing isoform 1 or 2 grew more rapidly than control cells expressing EGFP alone. However, to our surprise, cells expressing isoform 3 grew more slowly than cells expressing isoforms 1 or 2, or control cells (Figure 3). As previously, we ruled out that this was due to increased cell death because caspase-3 activity was the same in the 3 cell lines (data not shown). In addition cell cycle analysis confirmed these observations. In fact, we found the S/G1 phase ratio higher in cells expressing isoforms 1 or 2 than in control cells, and slower in cells expressing isoform 3 (Figure 4). Therefore, WSB1 isoforms 1 or 2 and isoform 3 have opposite effects on cell proliferation, isoform 3 inhibiting pancreatic cell growth whereas isoforms 1 and 2 stimulate their proliferation.

### WSB1 isoform 3 reduces susceptibility to stress-induced cell death

**Figure 5:**
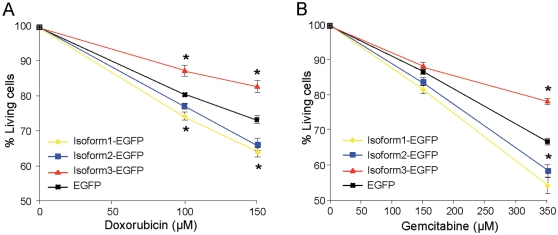
WSB1 isoform 3 reduces susceptibility to stress-induced cell death. Mia-PaCa2 cells were transduced with retrovirus expressing WSB1 isoforms 1, 2 or 3 as EGFP fusion protein or EGFP as control and selected with puromycin. Cells were treated with gemcitabine (100 and 150 µM) for 48 hours or with doxorubicin (150 and 350 µM) for 6 hours. Cell viability was measured by MTT. Values are expressed as the mean±S.D. of combined results from two independent experiments performed in triplicate. (*, p<0.05).

Since expression of isoform 3 is induced by stress, we investigated whether it could affect the susceptibility of the cells to stress-induced cell death. We submitted Mia-Paca2 cells expressing isoforms 1, 2 or 3 to treatment with 100 or 150 µM doxorubicin known to induce apoptosis, and measured cell viability after 6 h. We observed with both drug concentrations increased cell death in cells expressing isoforms 1 or 2, compared to control cells. On the contrary, cells expressing isoform 3 were significantly more resistant than controls (Figure 5). To check these findings, cells were treated for 48 h with 150 and 350 µM gemcitabine and cell viability was assessed. A profile similar to that of doxorubicin-treated cells was obtained, cells expressing isoforms 1 and 2 being more sensitive to the drug than control cells, contrary to cells expressing isoform 3 (Figure 5). These drugs kill dividing but not arrested cells, but the differences after 6 hour of treatment are too important to be totally explained by the fact that slower growing cells die more slowly. These results show that over-expression of WSB1 isoform 3 and down-regulation of isoforms 1 and 2 in response to cellular stress results in increased resistance to apoptosis.

## Discussion

The hypothesis supporting this work is that pancreatic tumor progression and metastasis occurs only when circulating cancer cells can successfully resist the stresses generated by the new microenvironment provided by the host tissue. If escaped cancer cells were not submitted to stress in new environments, metastasis would develop in all tissues. In fact, clinical observation is that primary cancers developing in a given organ always develop metastases in the same other organs, probably the ones in which these cancer cells are able to resist the stress caused by the environmental conditions. Consequently, therapeutic strategies that interfere with the mechanisms protecting cancer cells from stress might slow down or prevent the metastatic process. The first step towards establishing such strategies is to identify the genes that confer resistance to the new microenvironment during tumor development. This can be achieved by comparing by DNA array gene expression in cells growing in different microenvironmental conditions, then select the genes overexpressed in conditions of tumor growth. To mimic the stress that occurs during metastasis formation in patients, we used an experimental set-up based on a xenograft pancreatic tumor model. We compared the gene expression profiles of human pancreatic cancer cell lines growing in vitro under standard culture conditions (microenvironment A) and during tumor development after injection into nude mice (microenvironment B). Indeed, many genes showed overexpression in the new microenvironment and if some of them might be overexpressed in response to the signals like cell-to-cell or cell-to-extracellular matrix contact, others might be implicated in resistance to stress. We selected as best candidates the genes strongly overexpressed in the xenografts generated by all 5 cell lines. Among candidate genes, we chose to characterize in detail WSB1 because it presented the further interest of generating by alternative splicing three distinct isoforms whose regulation upon exposure to stress is not coordinated. Although over-expression of isoforms 1+2+3 is moderate, 1.75 to 3.93 fold depending of the cell type, isoform 3 is strongly over-expressed (3.7 to 9 fold). On the contrary, expression of isoforms 1+2 is decreased to 5 to 20% in xenografts. Interestingly, isoform 3 is also largely predominant in human pancreatic tumors, which supports our working hypothesis.

Although the U133A genechip has been designed to target human sequences, we cannot rule out a potential cross-hybridization of some probes with mouse sequences. This is why designed *Homo sapiens*-specific primers for qPCR. qPCR were performed on mouse embryonic fibroblasts cDNA in the same conditions as for human pancreatic cDNA and there was no DNA amplification after 45 cycles, although we were able to amplify the entire isoform 3 from the same mouse embryonic fibroblasts cDNA by a PCR with primers matching perfectly sequences common to both species (Forward 5′-CCATGAGCCAGCTTTCCC-3′; Reverse 5′-TCACACCAATATTGCTGCCAC-3′). Therefore, we concluded that our qPCR primers are human-specific and that the enhanced expression of isoform 3 occurs in human epithelial cells and not in mouse stroma.

To validate that WSB1 is indeed a stress-induced gene, we submitted pancreatic cells to several stresses and observed that expression of WSB1 isoform 3 was always strongly activated whereas expression of isoforms 1+2 was unchanged or decreased. That cellular stress regulates WSB1 expression by modulating its splicing is indeed interesting but not original since such regulation was already described for other genes. For example, modulations of alternative splicing occur for Arabidopsis SR protein homologues, atSR30 and atSR45a, in response to environmental stress [12]. Also, UV and γ radiation as well as cisplatin treatment induce alternative splicing of hdm2 [13] and exposure of primary lymphocytes to a stress induces the appearance of TSG101 splicing variants [14]. Finally, in neurons, acetylcholinesterase shifts from isoform AChE-S to the normally rare isoform AChE-R in response to several stresses [15]. The important point is that stress-induced modulation by alternative splicing of the expression of WSB1 isoforms is very similar to what we observed during xenograft tumor progression.

It is noteworthy that human WSB1 isoform 3 sequence has been permanently suppressed from databases for being a nonsense-mediated mRNA decay (NMD) candidate. NMD is an mRNA surveillance pathway that ensures the rapid degradation of mRNAs containing premature translation termination codons, thereby preventing the accumulation of truncated and potentially harmful proteins [16]. In this study, we show an accumulation of isoform 3 mRNA after stress induction, in xenograft and in human pancreatic tumors contrary to what would be expected in a NMD context. Therefore, although its sequence could be related to NMD, it is obvious that WSB1 isoform 3 mRNA is not rapidly degraded and does not seem to be a real target for NMD. We also ruled out by RT-PCR amplification and sequencing the entire isoform 3 mRNA the fact that a pre-mRNA is detected instead of isoform 3 mRNA (data not shown).

The physiological consequences for pancreatic cancer cells of stress-induced changes in the proportions of WSB1 isoforms, in terms of growth rate and resistance to apoptosis, are important. Expression of WSB1 isoforms 1 or 2 was shown to accelerate cell cycle whereas expression of isoform 3, on the contrary, slowed it down. When all isoforms were concomitantly targeted by a siRNA in Mia-PaCa2 or Panc1 cells growth was significantly reduced, which was expected because isoforms 1 and 2 are abundant in non-stressed cells that show normal growth. Hence, in stress conditions growth is limited both by the decreased expression of isoforms 1 and 2 and by the overexpression of isoform 3. On the other hand, expression of isoform 1 or 2 increases the sensitivity of cells to pro-apototic stimuli whereas expression of isoform 3 increases their resistance. Again, the stress-induced inhibition of isoforms 1 and 2 expression and overexpression of isoform 3 both help the cells coping with a different environment. The molecular mechanisms by which these proteins can affect cell growth and resistance to apoptosis in opposite directions remain to be determined, although the loss in isoform 3 of the C-terminal part of isoform 1 should probably account for part of it. Yet, in spite of important structural differences, the 3 WSB1 isoforms are similarly distributed in the cytoplasm as shown in Figure 1.

In conclusion, we report that the shift in WSB1 isoform expression observed in human pancreatic cancer cells submitted to a stress is similar to the shift observed when these cells are xenografted in nude mice or in human pancreatic tumors. If that shift results in decreased growth rate of the cells, it also increases significantly their resistance to apoptosis. These data support the hypothesis that activation of stress-response mechanisms helps cancer cells establishing metastases, inasmuch as the same WSB1 isoform ratio is observed in pancreatic adenocarcinomas. Whether the stress-induced changes in WSB1 isoform expression is necessary for tumor development, which would make it a potential therapeutic target, remains however to be investigated.

## Materials and Methods

### Cell lines and cell culture conditions

The human pancreatic cancer-derived Mia-PaCa2, BxPC3, Panc-1, Capan-1 and Capan-2, and the Phoenix Amphotropic viral packaging cell lines were cultivated as recommended by American Type Culture Collection.

### Subcutaneous tumor induction in nude mice

Suspensions of pancreatic cell lines, (10^7^/200 µl PBS) were injected subcutaneously into the flank of male athymic 7–8 week-old nude mice, and tumors were allowed to develop for 30 days. All studies were performed in accordance with the European Union regulations for animal experiments.

### Human pancreatic tumor samples

Pancreatic tissue was obtained from patients that underwent pancreatic resection at the Department of Surgery, Hopital Nord, Marseille, France. Institutional Review Board approval was obtained from the Comité d'Ethique de l'Institut Paoli Calmettes (Marseille, France). Written informed consent was obtained from the patients in accordance with the Helsinki Declaration.

### RNA isolation

Total RNA was isolated by the Trizol (Gibco-BRL) procedure.

### Microarray

The experiment was performed on U133 2.0 Plus Genechip (Affymetrix, Santa Clara, CA), as previously described[17] , and analyzed with the Microarray Suite 5.0 software (Affymetrix).

### Treatments

In some experiments, Mia-Paca2 cells were treated during 24 h with 10 µM Doxorubicin (Sigma), 0.5 µM Staurosporine (Sigma) or 350 µM Gemcitabine (Eli Lilly) or submitted to 24 h serum deprivation, or to γ-irradiation (5Grays).

### Hypoxia/Anoxia

Oxygen concentrations were modified using an hypoxia incubator chamber (catalogue # 27310; Stem Cell Technologies) and appropriate gas mixtures (Linde gas, France), following the instructions of the manufacturer. Cells were submitted to 5 h anoxia (5%CO_2_, 95% N_2_) or to 6–18 h hypoxia (5%CO_2_, 94.8% N_2_ and 0.2%O_2_).

### Quantitative RT-PCR analysis

First-strand cDNA was synthesized using Expand Reverse Transcriptase (Roche, Meylan, France). Quantitative PCR was performed using Roche Light Cycler system and reagents, following instructions of the manufacturer. Primers used and conditions of annealing were 5′-AGTGTCAGACTGATCTGG-3′ and 5′-ACAGTAGACGGATTCTTCAA-3′, 58°C for 7 sec; 5′-GATCATACTGAAGTGGTCAG-3′ and 5′-GCCCAAATTCCATCAGAATG-3′, 56°C for 14 sec; and 5′-GATCGTGGTTAGTTTGGG-3′ and 5′-TGGGTCACCAACTTGAG-3′, 57°C for 6 sec ; for global expression of wsb1, isoforms 1+2 and isoform 3 respectively. (see Supplementary Figure S1) Each sample was analyzed in duplicate and the experiment was repeated twice. Results were analyzed using RelQuant (Roche) and expressed as a ratio of Tata box Binding Protein (TBP) or of control values (untreated cells).

### Transfections

Cells were transfected using FuGENE HD (Roche Diagnostics) following the manufacturer's instructions with the pEGFP-N1 plasmid (Clontech) containing full-length human WSB1 cDNA, isoform 1, 2 or 3 (respectively NM_015626, NM_134265, NM_134264), or no insert. The 3 isoforms were also subcloned in the pcDNA4-V5-A plasmid (Invitrogen).

### WSB1 fluorescence

The mouse anti-V5 antibody (InVitrogen) was used to detect V5-tagged WSB1 proteins, and revealed by the goat chromeo 488-conjugated anti-mouse secondary antibody (ActiveMotif). Cells expressing the EGFP-WSB1 fusion proteins were only fixed. Cells were mounted in ProLong Gold (Invitrogen) and analyzed with a Nikon I90 microscope (40×/APO plan/NA1.40).

### Retroviral vector and retroviral-mediated gene transfer

The 3 isoforms of WSB1 fused to EGFP, or EGFP alone, were subcloned into the pLPCX retroviral vector obtained from S. Lowe (Cold Spring Harbor Laboratory, NY). Phoenix Amphotropic packaging cells were transfected with polyethyleneimine with 5 µg of retroviral plasmid. Forty-eight hours later, the supernatant was supplemented with 4 g/ml polybrene (Sigma) and added to the culture medium (v/v) of Mia-PaCa2 cells. The population of WSB1-EGFP-expressing cells was selected with 1 mg/ml puromycin. Mia-PaCa2 cells infected with the EGFP expressing vector were used as control.

### siRNA-mediated knock-down of WSB1 expression

WSB1 expression was knocked-down in cultured cells with a specific siRNA. Mia-PaCa2 cells were transfected using Xtremgene reagent (Roche Diagnostics), following the manufacturer's instructions, with a siRNA designed for WSB1 mRNA (sense 5′-GAAAACUCCUCCUUAACUUd(TT)-3′) (Dharmacon) or control siRNA, (sense 5′-UUCUCCGAACGUGUCACGUd(TT)-3′) (Qiagen).

### MTT/cell viability assays

Every day after siRNA transfection, cells were incubated with 0.5 mg/ml MTT (3-[4,5-dimethylthiazol-2-yl]-2,5-diphenyl tetrazolium bromide; Sigma) for 2 h at 37°C and 5% CO2. The reaction was stopped, and formazan crystals were dissolved in a 0.1N HCl-isopropanol solution. MTT metabolism (at an optical density of 570 nm [OD570]) was read and normalized to cell density (OD690). Assays were performed thrice and in triplicate. Alternatively, cell viability was determined by direct counting via trypan blue exclusion. The number of viable cells after treatment with gemcitabine during 48 h or doxorubicin during 6 h was determined by the trypan blue dye exclusion test.

### BrdU proliferation assays

Cell proliferation was measured using 5-Bromo-2′-deoxy-uridine Labeling and Detection Kit III (Roche Diagnostics) according to the instructions of the manufacturer, after 16 h incubation with BrdU. Results were normalized for cell viability (MTT).

### Caspase 3/7 activity

Caspase activity was measured in Mia-PaCa2 cells by using the Apo-ONE Homogeneous Caspase 3/7 Assay Fluorometric Kit (Promega), and performed as recommended by the manufacturer, 48 hours after transfection with WSB1 or control siRNA. Results were normalized for cell viability (MTT).

### Cell cycle analysis

Cells were synchronized by 24 h serum-starvation and collected after 24 h incubation in serum-containing DMEM. Then, cells were washed with PBS and fixed in cold-ethanol 70% for 30 minutes at 4°C. After a wash with PBS, cells were treated with 50 µL RNase A (100 µg/µL), labeled with 250 µL propidium iodide (50 µg/µL), and immediately analyzed by flow cytometry (FACSCalibur, Becton Dickinson, Le Pont-De-Claix, France). Cell cycle analysis was done on 25,000 cells, evaluating the G1, S and G2 phases (FlowJo 7.2.2). The experiments were repeated thrice and each sample was assayed in triplicate.

### Statistical analysis

Statistical analysis was performed by ANOVA with post hoc analysis Student-Neuman-Keuls test. Results shown represent mean±S.D.

## Supporting Information

Figure S1 Schematic representation of each isoform mRNA and location of the primers used for qPCR.
(0.50 MB TIF)

Figure S2 WSB1 expression in stable cell lines. Expression of each transfected WSB1 isoform as EGFP fusion protein was evidenced by western blot, using an anti-EGFP antibody.
(0.14 MB TIF)
