# Supplementary figures and images for: The WSB1 Gene Is Involved in Pancreatic Cancer Progression
Source: PLoS One. 2008 Jun 25;3(6):e2475. doi: 10.1371/journal.pone.0002475 (PMC2423480; doi:10.1371/journal.pone.0002475)

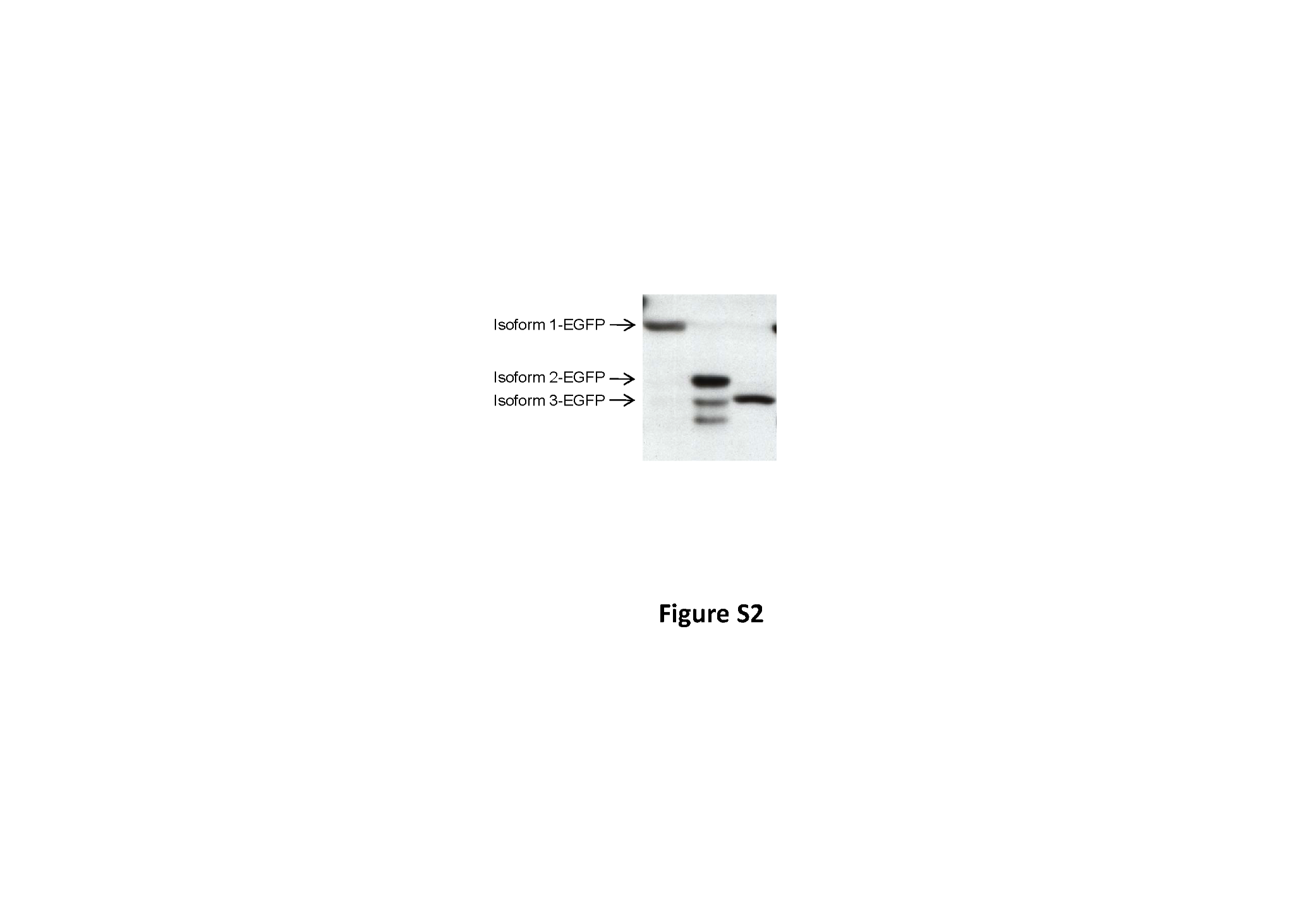

Supplement: Figure S2 — WSB1 expression in stable cell lines. Expression of each transfected WSB1 isoform as EGFP fusion protein was evidenced by western blot, using an anti-EGFP antibody. (0.14 MB TIF) [file pone.0002475.s002.tif]
